# ARHGEF15 overexpression worsens the prognosis in patients with pancreatic ductal adenocarcinoma through enhancing the motility and proliferative activity of the cancer cells

**DOI:** 10.1186/s12943-016-0516-4

**Published:** 2016-05-04

**Authors:** Hiroto Fukushima, Makiko Yasumoto, Sachiko Ogasawara, Jun Akiba, Yuhei Kitasato, Masamichi Nakayama, Yoshiki Naito, Yusuke Ishida, Yoshinobu Okabe, Masafumi Yasunaga, Hiroyuki Horiuchi, Etsuko Sakamoto, Hiraku Itadani, Shinji Mizuarai, Shinji Oie, Hirohisa Yano

**Affiliations:** Biomarker Research, Discovery and Preclinical Research Division, Taiho Pharmaceutical Co., Ltd., 3 Okubo, Tsukuba, Ibaraki 300-2611 Japan; Department of Pathology, Kurume University School of Medicine, 67 Asahi-machi, Kurume, Fukuoka 830-0011 Japan; Division of Gastroenterology, Department of Medicine, Kurume University School of Medicine, 67 Asahi-machi, Kurume, Fukuoka 830-0011 Japan; Department of Surgery, Kurume University School of Medicine, 67 Asahi-machi, Kurume, Fukuoka 830-0011 Japan

**Keywords:** Pancreatic ductal adenocarcinoma, ARHGEF15, Prognostic marker, Rho

## Abstract

**Background:**

Pancreatic ductal adenocarcinoma (PDAC) is one of the most aggressive neoplastic diseases, associated with a remarkably poor prognosis. However, the molecular mechanisms underlying the development of PDAC remain elusive. The aim of this study was to identify genes whose expressions are correlated with a poor prognosis in PDAC patients, and to unravel the mechanisms underlying the involvement of these genes in the development of the cancer.

**Methods:**

Global gene expression profiling was conducted in 39 specimens obtained from Japanese patients with PDAC to identify genes whose expressions were correlated with a shorter overall survival. The effect of gene silencing or overexpression of ARHGEF15 in pancreatic cancer cell lines was examined by introducing siRNAs of ARHGEF15 or the ARHGEF15 expression vector. After assessing the effect of ARHGEF15 deregulation on the Rho-family proteins by pull-down assay, wound healing, transwell and cell viability assays were carried out to investigate the cellular phenotypes caused by the perturbation.

**Results:**

The global mRNA expression profiling revealed that overexpression of ARHGEF15, a Rho-specific GEF, was significantly associated with a poor prognosis in patients with PDAC. We also found that the depletion of ARHGEF15 by RNA interference in pancreatic cancer cell lines downregulated the activities of molecules of the Rho signaling pathway, including RhoA, Cdc42 and Rac1. Then, we also showed that ARHGEF15 silencing significantly reduced the motility and viability of the cells, while its overexpression resulted in the development of the opposite phenotype in multiple pancreatic cancer cell lines.

**Conclusion:**

These data suggest that upregulation of ARHGEF15 contributes to the development of aggressive PDAC by increasing the growth and motility of the pancreatic cancer cells, thereby worsening the prognosis of these patients. Therefore, ARHGEF15 could serve as a novel therapeutic target in patients with PDAC.

**Electronic supplementary material:**

The online version of this article (doi:10.1186/s12943-016-0516-4) contains supplementary material, which is available to authorized users.

## Background

Pancreatic ductal adenocarcinoma (PDAC) is one of the most aggressive cancers, and is the fourth most frequent cause of cancer-related death in both Japan and the US. The prognosis of patients with this disease is extremely poor, with an overall median survival of 5 to 8 months. Since it is difficult to diagnose pancreatic cancer at an early stage, approximately 80 % of patients with PDAC have unresectable disease, including locally advanced or distant metastatic disease, at diagnosis [1, 2]. Therefore, clarification of the molecular mechanism underlying the exceptionally poor prognosis of PDAC, and identification of novel therapeutic targets for the treatment of PDAC are urgently needed.

The Rho-family GTPases are important intracellular signaling molecules that regulate cytoskeleton organization, gene expressions, cell cycle progression, cell motility and other cellular processes through modulating the activities of the downstream molecules, including p21-activated kinase (PAK), Rho-kinase and the myosin-binding subunit of myosin phosphatase [3]. Abnormal Rho GTPase activities have been implicated in multiple human pathologies [4, 5]. RhoA, in particular, is activated in several human cancers and is reported to be involved in cancer progression and metastasis [6–9]. The Rho-family small GTPases cycle between the GTP-bound form, which is the active form, and the GDP-bound form, which is the inactive form, in response to several upstream signals, including growth factors, cytokines, adhesion molecules, etc. These signals activate guanine nucleotide exchange factors (GEFs), which in turn activate small GTPases by promoting the loading of GTP on to the small GTPases, a rate-limiting step in GTPase regulation [10, 11]. Accumulated reports have shown that the GEFs for the Rho-family proteins are deregulated in multiple types of cancers. VAV guanine nucleotide exchange factor 1 (VAV1) is reported to be overexpressed in clinical pancreatic carcinoma cells, leading to activation of Rac1 signaling, resulting in decreased survival in pancreatic cancer patients [12, 13]. VAV guanine nucleotide exchange factor 2 (VAV2) is hyperactivated in head and neck squamous cell carcinoma, and its molecular role was assessed by VAV2-silencing; this investigation revealed that inactivated Rac1 signaling leads to a decreased invasiveness of cancers [14]. Another example signifying the crucial roles of GEFs in cancer development is the identification of the chromosomal rearrangement in acute myelogenous leukemia (AML) that results in the generation of the fusion protein MLL-ARHGEF12 [15]. These reports suggest the potentially significant roles of RhoGEFs in tumorigenesis.

Rho guanine nucleotide exchange factor 15 (ARHGEF15) has been reported to function as a Rho-specific GEF. Recently, an additional function of ARHGEF15 was found: disruption of the ARHGEF15 gene led to delayed extension of vascular networks and consequent reduction of the total vessel area in the retina [16]. However, there have been only a few reports on the significance of ARHGEF15, and the precise functions of ARHGEF15 in PDAC remain elusive.

In this study, we attempted to identify genes whose expressions are correlated with a poor prognosis in patients with PDAC by global expression microarray analysis of clinical samples, and investigated how the identified genes were involved in the development of cancer at the molecular level. At the outset, we found that ARHGEF15 was overexpressed in the tumors in PDAC patients with a poor prognosis. In addition, ARHGEF15 was shown to facilitate cell growth and cell motility in PDAC cell lines. Our data indicated that ARHGEF15 promotes the development of the aggressive features of PDAC, and could serve as a biomarker for assessing the aggressiveness and predicting the prognosis of PDAC patients, and also serve as a potential target for the development of treatments directed against PDAC.

## Results

### Clinicopathologic characteristics of PDAC patients

Tumor samples from a total of 39 patients with pancreatic ductal adenocarcinoma (PDAC) were collected between 2002 and 2010, and subjected to molecular profiling using DNA microarrays. Details of the clinical characteristics of the patients are presented in Table 1. Among the PDAC patients, there were 27 male and 12 female patients, respectively, with an overall median age of 64.5 years (range, 42 to 78 years). The median overall survival (OS) was 17.5 months (range, 1.7 to 62.8 months), and the pathological stage ranged from I to IVb. No statistically significant relationship was found between the OS and any of the patient characteristics, including the gender, age, tumor histology, tumor size, tumor location or the pathologic stage (Table 1, *p* > 0.05, log rank test).

**Table 1:** Patient characteristics

Characteristic		*P* value
Patient number	*N* = 39	
Gender		0.48
Female	12	
Male	27	
Age (years)		0.63
Median	64.5	
Range	42-78	
Histology(differentiation)		0.13
Well	12	
Moderate	21	
Poor	3	
Other	3	
Primary tumor size(mm)		0.38
Median	28	
Range	10–90	
Location of tumor		0.27
Pb	8	
Ph	24	
Pt	6	
Phb	1	
Clinical stage		0.78
I	1	
II	4	
III	18	
IV a	11	
IV b	5	

*Abbreviations*: *Pb* Pancreatic body, *Ph* Pancreatic head, *Pt* Pancreatic tail, *Phb* Pancreatic head and body

### The ARHGEF15 expression level was elevated in the tumor tissues of PDAC patients with a poor Prognosis

**Fig. 1:**
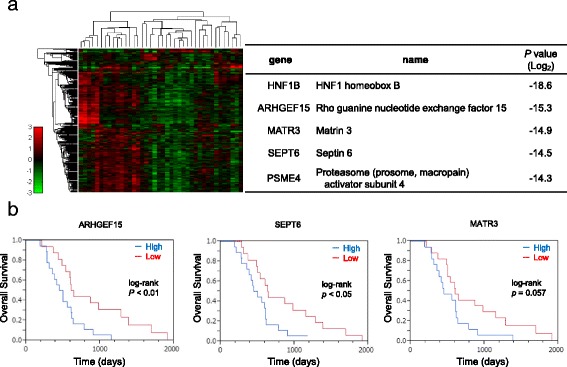
High ARHGEF15 expression levels in pancreatic cancers are associated with a poor prognosis. **a** A heatmap of differentially expressed genes was determined by Affymetrix X3P GeneChip microarray analysis in specimens obtained from 39 PDAC patients. The top five most differentially expressed genes between the poor prognosis and better prognosis groups are listed. **b** Kaplan-Meier estimates of the relationships between the expression levels of ARHGEF15, SEPT6 and MATR3 in the tumors by separate real-time RT-PCR analyses and the prognosis of the PDAC patients

To identify the molecular mechanism underlying the exceptionally poor prognosis of PDAC, we performed a global gene expression analysis using the Affymetrix X3P GeneChip microarray, and carried out an analysis of the prognosis in relation to the tumor gene expression profiles of the 39 PDAC patients enrolled in the study. The samples were divided into two groups: the poor prognosis group, in which the OS was shorter than the median OS, and the better prognosis group, in which the OS was longer than the median OS. We found that the expressions of 46 genes were significantly correlated with the OS in the PDAC patients (Additional file 1: Table S1, *p* < 0.0001). Of the 46 genes, 32 were overexpressed in the worse prognosis group, whereas 14 were overexpressed in the better prognosis group (Fig. 1a). The top five most differentially expressed genes between the poor prognosis group and better prognosis group were HNF1B, ARHGEF15, SEPT6, MATR3 and PMSE4. The gene showing the most significant differential expression was HNF1B (Additional file 2: Figure S1, *p* < 0.00001), known as a transcription factor, whose higher expression level in the PDAC specimens was correlated with a worse survival of the patients [17]. On the other hand, the relationships between the other 4 genes and the prognosis of the patients with PDAC have not been reported to date.

To verify the differential expressions of the genes identified by microarray analysis, we performed independent real-time RT-PCR analyses for ARHGEF15, SEPT6, MATR3, and PMSE4. In the RNA samples obtained from the 39 formalin-fixed tumors that were analyzed, reliable data were obtained for 35 samples, as judged from the expression of ACTB, the internal control gene. Real-time RT-PCR analysis showed higher expression levels of the ARHGEF15, SEPT6 and MATR3 genes among the four genes in the worse prognosis group as compared to the better prognosis group. The results were analyzed by constructing Kaplan-Meier plots and compared by the log-rank test (Fig. 1b). In particular, the patient with higher expression levels of ARHGEF15 showed a significantly shorter OS as compared to the group showing lower ARHGE15 expression levels (median OS 14.0 months in the high ARHGEF15 expression group vs. 26.2 months in the low ARHGEF15 expression group; *p* < 0.01, log rank test). These data indicated that ARHGEF15 upregulation might enhance the aggressiveness of PDAC cells, resulting in a worse prognosis of the patients.

### Analysis of ARHGEF15 expression in a pancreatic cancer cell line panel

**Fig. 2:**
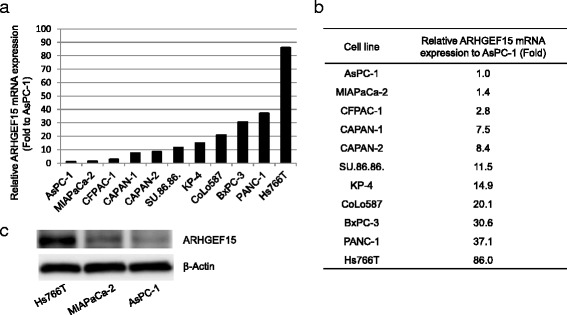
Varied ARHGEF15 expression levels in the pancreatic cancer cell lines. The ARHGEF15 mRNA expression levels were determined in 11 pancreatic cancer cell lines by real-time RT-PCR (*n* = 3). The relative gene expression levels of ARHGEF15 represented in fold values relative to the minimum expression level in the AsPC-1 cells (**a** and **b**). **c ** The protein expression levels of ARHGEF15 in Hs766T, MIAPaCa-2 and AsPC-1 cells were determined by Western blotting (*n* = 3)

Since ARHGEF15 is a specific GEF for the Rho-family proteins that is involved in multiple cancer signaling pathways, we further investigated the molecular role of ARHGEF15 in the development of PDAC using pancreatic cancer cell lines. We initially assessed the ARHGEF15 expression levels in 11 pancreatic cancer cell lines by real-time RT-PCR analysis. As shown in Fig. 2a and b, varied expression levels were observed in the cell lines, with a maximum difference in the ARHGEF15 expression level of approximately 86-fold among the pancreatic cancer cell line panel. While the AsPC-1 and MIAPaCa-2 cells showed lower ARHGEF15 mRNA expression levels, the Hs766T cells showed the highest expression levels. ARHGEF15 protein expression levels in the three cell lines were also examined by Western blotting, which revealed expression patterns consistent with the mRNA expression patterns (Fig. 2c).

### Gene silencing of ARHGEF15 decreased the amounts of the activated forms of the Rho-family proteins

**Fig. 3:**
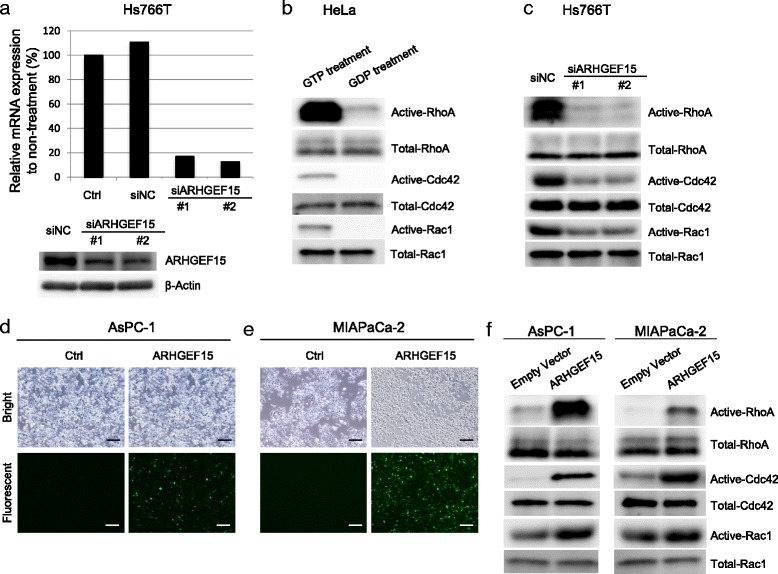
ARHGEF15 expression is associated with activation of the Rho-family proteins in several cell lines. **a** ARHGEF15 expression levels in untreated Hs766T cells (Ctrl), Hs766T cells treated with negative control siRNA (siNC) or siRNA for ARHGEF15 were assayed by both real-time RT-PCR and Western blotting. **b** Confirmation by the active RhoA/Cdc42/Rac1 pull-down assay. HeLa cell lysates treated with GDP (to inactivate the Rho-pathway proteins) or GTP (to activate the Rho-pathway proteins) were subjected to pull-down assay for the active Rho-family proteins to examine the degree of activation of the Rho-family proteins. **c** RhoA, Cdc42 and Rac1 activation in Hs766T cells after knockdown of endogenous ARHGEF15 measured by the active RhoA/Cdc42/Rac1 pull-down assay. Overexpression of ARHGEF15 in **d** AsPC-1 cells and **e** MIAPaCa-2 cells. RhoA, Cdc42 and Rac1 activation in **f** AsPC-1 cells and **g** MIAPaCa-2 cells transfected with the ARHGEF15 expression vector assessed by the active RhoA/Cdc42/Rac1 pull-down assay (*n* = 3). The *scale bars* represent 200 μm

Since higher ARHGEF15 expression levels were associated with a worse prognosis, we attempted to investigate the biological effects of overexpression or knockdown of ARHGEF15 on the cellular phenotype using pancreatic cancer cell lines. First, we examined the effect of gene silencing of ARHGEF15 by RNA interference in the Hs766T cells, which showed the highest endogenous expression levels of ARHGEF15 among the pancreatic cell line panel, as shown in Fig. 2a. We confirmed by real-time RT-PCR that small-interfering (si) RNAs for ARHGEF15 (siARHGEF15s) suppressed the ARHGEF15 mRNA expression level by more than 80 % as compared to the control cells (Fig. 3a). ARHGEF15 protein expression was also examined by Western blotting, which revealed expression patterns consistent with the mRNA expression patterns. Given the fact that the Rho-family proteins are direct downstream effectors of ARHGEF15, the amounts of activated RhoA, Rac1 and Cdc42 were determined in the ARHGEF15-silenced cells; pull-down assay was conducted to measure the amounts of GTP-bound activated RhoA, Rac1 and Cdc42. Lysates of HeLa cells treated with GTP as positive controls showed increased amounts of the activated forms of the Rho-family proteins, while the lysates of the cells treated with GDP as negative controls showed decreased amounts of the activated forms of the Rho-family proteins, confirming the specificity of the assay (Fig. 3b). ARHGEF15 gene silencing in Hs766T cells reduced the amounts of activated GTP-RhoA, -Rac1 and -Cdc42 at 72 h (Fig. 3c and Additional file 3: Figure S2a). Then, we investigated the effect of ARHGEF15 overexpression on the amounts of the activated forms of the Rho-family proteins. Both AsPC-1 and MIAPaCa-2 cells, which showed low endogenous expression levels of ARHGEF15, were transiently transfected with an expression vector encoding ARHGEF15. Detection of the Halo tag attached to the ARHGEF15 protein confirmed that more than 50 % of the cells overexpressed the exogenous ARHGEF15 (Fig. 3d and e). Pull-down assay for active Rho-family proteins showed that the levels of GTP-bound active RhoA, Rac1 and Cdc42 were significantly increased in the AsPC-1 cells that showed ARHGEF15 overexpression (Fig. 3f). ARHGEF15 overexpression in MIAPaCa-2 cells was also associated with a tendency towards increase in the amounts of active Rho-family proteins. Collectively, the results indicate that both gene silencing and overexpression of ARHGEF15 modulate the degree of activation of the Rho-family proteins.

### ARHGEF15 promotes cancer cell motility

**Fig. 4:**
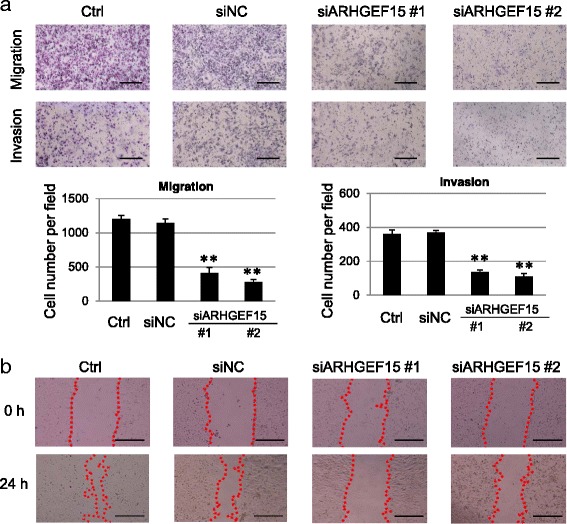
ARHGEF15 depletion decreases the cellular motility. **a** Cell migration and invasiveness of Hs766T cells (*n* = 4) following knockdown of ARHGEF15 were examined by the transwell chamber assay. The *scale bars* represent 500 μm. Graphs below the pictures indicate the numbers of the migrated cells. Statistical analysis was conducted by Student’s *t*-test as shown (**p <0.01). **b** Scratch wound healing assay in Hs766T cells after ARHGEF15 gene silencing (*n* = 4). *Dotted lines* represent the cell fronts at the gaps. The *scale bars* represent 500 μm

**Fig. 5:**
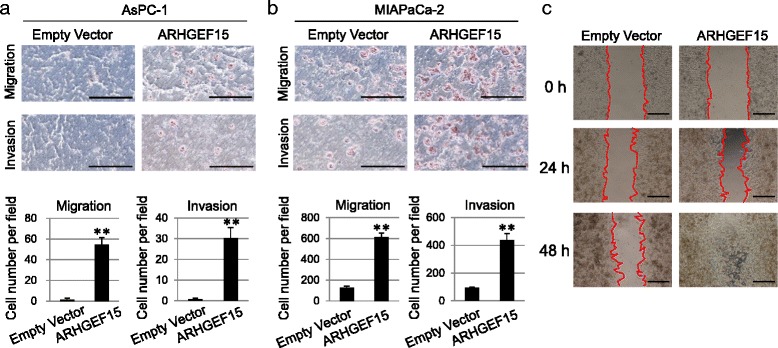
ARHGEF15 overexpression enhances the cellular motility. **a** and **b** Cell migration and invasiveness assay of AsPC-1 and MIAPaCa-2 cells (*n* = 4) following overexpression of ARHGEF15 was conducted using a transwell chamber. The *scale bars* represent 500 μm. The graphs below the pictures show the numbers of the migrated cells. Statistical analysis was conducted by Student’s *t*-test as shown (**p <0.01). **c** Scratch wound healing assay in MIAPaCa-2 cells showing ARHGEF15 overexpression (*n* = 4). *Dotted lines* represent the cell fronts at the gaps. The *scale bars* represent 500 μm

Rho-family GTPases are critical intracellular signaling molecules that regulate cytoskeleton organization, gene expression, cell cycle progression and cell motility [3]. Therefore, we examined whether activation of the Rho-family proteins by ARHGEF15 affected the aggressiveness of the pancreatic cancer. The effect of the ARHGEF15 expression level on the cellular motility was investigated using the transwell and wound healing assays. As shown in Fig. 4a, gene silencing of ARHGEF15 caused a notable reduction in the number of Hs766T cells, as compared to the control cells, migrating through the holes in the filters of the transwell chambers. In addition, we assessed the effect of ARHGEF15 gene silencing on the migration of cells into the scratch wound area at 24 h after generation of the wound in the presence of the cells. While almost complete closure of the entire wound area was observed in the control cells, the wound closure area decreased in the cells treated with siARHGEF15 (Fig. 4b). The retarding effect on wound healing and migration was confirmed again by using two different siRNA sequences for ARHGEF15. In addition, we assessed the effect of ARHGEF15 overexpression on the motility of the AsPC-1 and MIAPaCa-2 cells by the transwell and wound healing assays. ARHGEF15 overexpression in both of these cell lines was associated with a significant increase in the number of migrating cells in both the transwell (Fig. 5a and b) and wound healing (Fig. 5c) assays, consistent with the phenotypes of ARHGEF15 gene silencing.

### ARHGEF15 is involved in pancreatic cancer cell proliferation

**Fig. 6:**
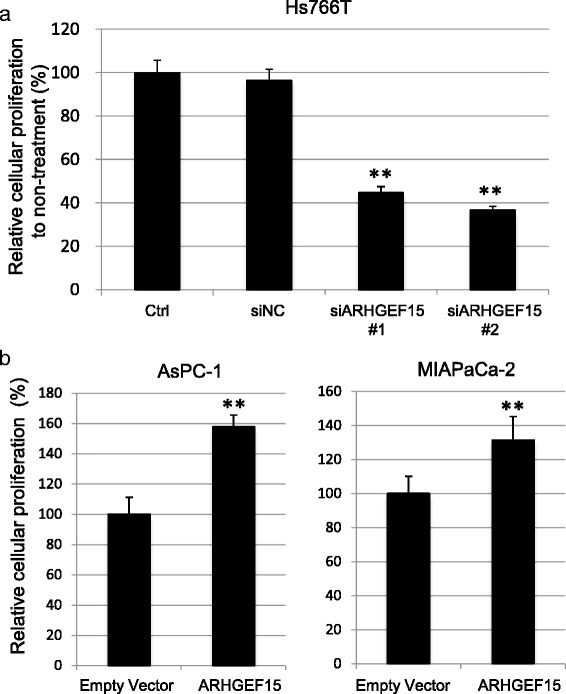
ARHGEF15 overexpression promotes cell growth. **a** Cell growth after knockdown of ARHGEF15 in Hs766T cells was examined at 72 h by a colorimetric modified MTT assay (*n* = 4). **b** Cell proliferation in response to ARHGEF15 overexpression was assessed at 72 h in AsPC-1 and MIAPaCa-2 cells by a colorimetric modified MTT assay (*n* = 4). Statistical analysis was conducted by Student’s *t*-test as shown (**p <0.01)

In addition to promoting cell motility, the Rho-family proteins are also critical intracellular signaling molecules that contribute to cell growth through associating with various proteins. We next examined whether modulation of ARHGEF15 expression affected the proliferation of pancreatic cancer cell lines, using Cell Counting Kit-8, a colorimetric modified MTT assay kit. First, we examined the effect of suppression of ARHGEF15 on the growth rate of Hs766T cells which were demonstrated to show high endogenous ARHGEF15 expression levels. As shown in Fig. 6a, the Hs766T cells treated with siARHGEF15s showed a 44.7 % and 36.7 % decrease of the cell proliferative activity at 72 h as compared to the controls. The decreased cell proliferation was confirmed by an independent time-course assay using a different siRNA for ARHGEF15 (Additional file 3: Figure S2a). Next, we assessed the effect of ARHGEF15 overexpression on the growth rate of the AsPC-1 and MIAPaCa-2 cells, which revealed an approximately 60 % increase in the proliferative activity of the AsPC-1 cells, and approximately 30 % increase in the proliferative activity of the MIAPaCa-2 cells at 72 h (Fig. 6b). The time-course study of ARHGEF15 overexpression also confirmed the effect of ARHGEF15 overexpression of enhancing the proliferative activity of the pancreatic cells (Additional file 3: Figure S2b). The results of the upregulation and downregulation experiments led us to infer that ARHGEF15 overexpression in the tumor contributes to the aggressiveness of PDAC.

**Fig. 7:**
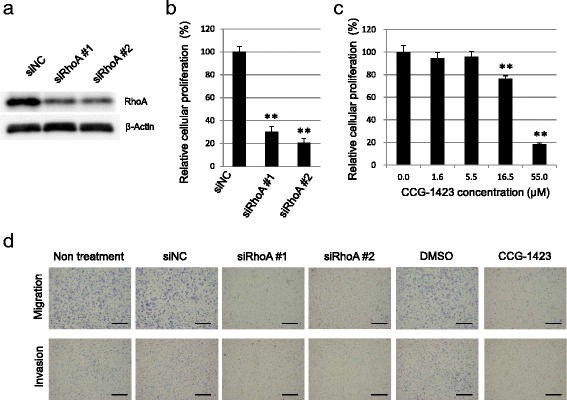
RhoA inhibition decreases both the proliferative activity and motility of the cancer cells. **a** RhoA expression levels in Hs766T cells treated with negative control siRNA (siNC) or siRNAs for RhoA were assayed by Western blotting. **b** and **c** Cell growth in Hs766T cells after knockdown of RhoA and treatment with the RhoA-specific inihibitor, CCG-1423, was examined at 72 h by a colorimetric modified MTT assay (*n* = 4). Statistical analysis was conducted by Student’s *t*-test as shown (**p <0.01). **d** Cell migration and invasiveness assays of Hs766T cells with gene silencing of RhoA and treated with 2.8 μM of CCG-1423 (*n* = 4) were conducted using a transwell chamber. The *scale bars* represent 500 μm

Finally, we assessed the effect of a RhoA inhibitor (CCG-1423) and RhoA gene silencing on the cellular phenotypes to obtain evidence in support of our findings. In accordance with the observation found in association with ARHGEF15 depletion, both the RhoA inhibitor and RhoA gene silencing reduced the proliferative activity, migration and invasiveness of the cancer cells (Fig. 7a–d).

## Discussion

Pancreatic ductal adenocarcinoma (PDAC) is one of the most aggressive cancers, and is associated with an extremely poor prognosis [1, 2]. We attempted to identify genes whose overexpression in the tumor might be correlated with a poor prognosis in PDAC patients by a global expression microarray analysis of clinical samples obtained from Japanese patients.

Although overexpression of several genes, including HNF1B, CA19-9 and miR-192, have been reported previously as poor prognostic factors in PDAC patients [17–19], we found for the first time that ARHGEF15 overexpression was also associated with an extremely poor prognosis of PDAC patients. Patients showing high expression levels of ARHGEF15 showed a statistically significantly shorter survival as compared to those showing low expression levels of ARHGEF15. Taking into consideration the numerous reports suggesting that the Rho-GEFs contribute to various steps of oncogenesis and to worsening of the prognosis of cancer patients [12, 13, 15, 20], it would be reasonable to conclude that ARFGEF15 worsens the prognosis of PDAC patients through facilitating migration and proliferation of the pancreatic cancer cells.

We showed that increased ARHGEF15 expression activated the Rho-family proteins, leading to enhanced cell motility in pancreatic cell lines. Molecular analysis to elucidate the mechanism by which activated Rho-family proteins increase the cellular motility was not conducted in the present study. However, the sequence of events from activation of the Rho-family proteins to increase of the cellular motility could be explained by the following well-established molecular mechanism reported previously; RhoA directly promotes phosphorylation of the regulatory myosin light chain, promotes organization of the actin stress fibers, and promotes the formation of focal adhesions [21]. This is consistent with the previous report that RhoA is activated at the leading edge of migrating cells [22]. Furthermore, Rho-family proteins bind several effector proteins, mediating downstream signaling. ROCK as one of the effector proteins facilitates contractility of muscle fibers and stress fiber formation [23]. Other examples of Rho effector proteins are mammalian homolog of *Drosophila diaphanous* (mDia) and phosphatidylinositide 4P 5kinase (PI4P-5 K) which enhance and promote reorganization of F-actin assembly in the filopodia [24, 25]. We showed that upregulation of ARHGEF15 in pancreatic cancer increased activation of the Rho-family proteins, especially RhoA, Cdc42 and Rac, resulting in enhanced motility of the pancreatic cancer cells. We speculate that the observed phenotypes related to motility in the study of ARHGEF15 dysregulation were mediated by the above-mentioned sequential molecular events resulting in the promotion of stress fiber formation.

In addition to the reduced cellular motility mediated by suppression of Rho signaling observed upon gene silencing of ARHGEF15, we found, unexpectedly, that ARHGEF15 also promoted the proliferation of the pancreatic cancer cells. However, several previous studies have demonstrated that overexpression of the Rho-family proteins together with enhanced Rho signaling was involved in the proliferation of cancer cells in many malignant tumors [14, 26–28]. Ghosh PM et al. reported that the PI3K pathway is involved in the enhancement of cellular proliferation induced by RhoA [29]. Zhang S et al. reported that RhoA activation is crucial for cell cycle progression of gastric cancer cells, and both activation of the RhoA-ROCK pathway and regulation of the CDKs are involved in the cell cycle regulation by RhoA [27]. On the other hand, recent observations have revealed that small-molecule inhibitors targeting RhoA, such as Rhosin and Y16, not only suppress the cellular motility, but also suppress the proliferative activity of cancer cells in vitro [30, 31]. Y-27632, another RhoA pathway inhibitor, was shown to cause cellular apoptosis in some cancer cell lines [32]. In addition to RhoA inhibitors, Cdc42-selective inhibitors have also been reported to decrease the cellular motility [33]. AZA1, an inhibitor of both Rac1 and Cdc42, was also found to suppress cellular proliferation both in vitro and in vivo [34]. Moreover, Kusuhara S et al. found that ARHGEF15 promotes retinal neovascularization [16]. These reports lend support to our findings that in addition to enhancing the motility and invasiveness of the cancer cells, ARHGEF15 also regulates the proliferation of pancreatic cancer cells.

We showed that depletion of ARHGEF15 in pancreatic cancer cells by small-interfering RNA caused inactivation of the Rho-family proteins, as shown in Fig. 3, resulting in suppression of both the motility and proliferative activity of the pancreatic cancer cells. Furthermore, we demonstrated that RhoA inactivation by gene silencing and RhoA inhibition (CCG-1423) suppressed both the cellular proliferative activity and motility of the cancer cells (Fig. 7a–d). These findings suggest that ARHGEF15 could serve as a therapeutic target for the development of treatments against pancreatic cancer, and that ARHGEF15 inhibition might have anti-cancer effects, including metastasis-inhibitory effect, anti-proliferative effect and anti-angiogenic effect, via suppressing activation of the Rho-family proteins. Accumulating evidence suggests that GEFs could be target proteins which can be inhibited by small molecules. Examples include a natural product named Brefeldin A, which targets the catalytic domain of GEF [35], and LM11 screened *in silico*, which binds to the GEF-GDP complex suppressing downstream signaling transduction [36]. These findings of chemical trackability together with our biological finding of the role of ARHGEF15 might pave the way for the development of treatments for patients with PDAC in the future. The effects of selective ARHGEF15 inhibition and also of ARHGEF15 inhibition on other tumor types remain to be determined in future studies.

## Conclusions

In summary, we found that overexpression of ARHGEF15 in PDAC patients was associated with enhanced growth and motility of pancreatic cancer cells, resulting in a significantly worse prognosis of the patients. Our findings in this study suggest that ARHGEF15 could serve as a novel target for the treatment of PDAC, especially benefiting the subpopulation of patients showing overexpression of ARHGEF15 in the tumor cells.

## Methods

### Tumor specimens

Formalin-fixed, paraffin-embedded (FFPE) specimens of pancreatic ductal adenocarcinoma were collected from patients (*n* = 39) at the Department of Pathology, Kurume University School of Medicine (Kurume, Japan), from 2002 to 2010. Ethical approval for the study was given by both the Kurume University Ethics Committee and the Research Ethics Review Committee of Taiho Pharmaceutical Co., Ltd. The permitted study numbers are 10203 at Kurume University and S10-010 at Taiho Pharmaceutical Co., Ltd. Informed consent was obtained from each patient prior to participation in the study. The present clinical study was carried out in compliance with the principles laid down in the Helsinki Declaration, as well as the guidelines of the institutional Ethics Committees. The patients’ prognoses were determined based on the clinical follow-up data obtained from the patients’ medical records, and the overall survival was measured from the day of surgery.

### Microarray analysis

All FFPE blocks were sectioned into 4-μm-thick sections with a Leica SM2010R microtome (Leica Microsystems K.K., Tokyo, Japan) using an RNase-free technique, and mounted on Superfrost slides. Two slides were prepared for each block: one stained with hematoxylin and eosin, and the other used for the subsequent RNA extraction. The tumor areas of the tissue sections were macro-dissected and RNA from these areas was isolated, linearly amplified and hybridized to the Affymetrix GeneChip Human X3P Array (Affymetrix, Santa Clara, CA, USA) using labeling methods, in accordance with the manufacturer’s instructions for the Arcturus Paradise PLUS Reagent System (Life Technologies, Grand Island, NY, USA) and GeneChip 3’ IVT Express Reagent kit (Affimetrix). Affymetrix array CEL files were processed by the RMA algorithm [37] to obtain probe set-level gene expression data, using the Expression Console software (Affymetrix). Hierarchical clustering of the microarray data was performed using the MATLAB software (The MathWorks, Natick, MA, USA). The R survival package was used for the survival analysis.

### Cell culture, plasmid, siRNA and RhoA inhibitor

The human pancreatic cancer cell lines AsPC-1, MIAPaCa-2, CFPAC-1, CAPAN-1, CAPAN-2, SU.86.86., BxPC-3, CoLo587, Panc-1 and Hs766T were purchased from American Type Culture Collection (ATCC, Rockville, MD, USA), while the KP-4 cell line was purchased from the Japanese Collection of Research Bioresources Cell Bank (JCRB Cell Bank, Osaka, Japan). Cells were cultured in the recommended media supplemented with fetal bovine serum and the required reagents. All cells were incubated at 37 °C in a humidified atmosphere containing 5 % CO_2_. The Halo-tagged ARHGEF15 expression vector pFN21A was purchased from Promega (Madison, WI, USA). The small-interfering RNAs (siRNA) against ARHGEF15 (ARHGEF15HSS117853 and ARHGEF15HSS117854) and RhoA (RHOAVHS40471 and RHOAHSS100655) were purchased from Life Technologies. CCG-1423, a RhoA-specific inhibitor, was purchased from Sigma-Aldrich (St. Louis, MO, USA).

### Cell viability assay

The cell viability was quantified by a colorimetric modified MTT assay using Cell Counting Kit-8 (Dojindo, Kumamoto, Japan), in accordance with the manufacturer’s instructions. Cells were seeded on to four wells of 96-well plates at a density of 5 × 10^2^ cells/well. The cell viability assay was performed 72 h after the seeding. Ten μL of Cell Counting Kit-8 was then added to each well. After incubation at 37 °C for 4 h, the absorption at 570 nm was measured using a microplate reader. The proliferative activities of the cells transfected with siRNA or plasmid were also quantified at 72 h after the transfection by a colorimetric assay.

### Transwell migration and invasiveness assay

Cell migration assays were performed using transwell chambers (24-well, 8-μm pore size; Corning, Corning, NY, USA) and cell invasiveness assays were conducted using BD Falcon Cell culture inserts coated with BD matrigel matrix (24-well, 8-μm pore size; BD Bioscience, San Jose, CA, USA). About 1 × 10^5^ cells in DMEM containing 0.5 % serum were loaded into the upper chambers. The lower chambers were filled with the same medium supplemented with 10 % FBS. The plates were incubated at 37 °C for 12 h. Cells that did not migrate through the pores were removed with a cotton swab. Cells on the lower side of the insert filter were fixed with 10 % formalin and stained with hematoxylin and eosin. The number of cells on the underside of the filter was counted.

### Wound-healing assay

Scratch wound-healing assays were performed in 24-well tissue culture plates (Corning). At 24 h after the cells were seeded (by which time, the cell confluence usually reached 90–100 %), scratches were made using the tip of a 200-μL pipette. The wells were then washed twice with the medium and cultured for an additional 24 or 48 h, followed by assessment of the wound area.

### Active RhoA, Cdc42 and Rac1 pull-down assay

Cells were grown in 10-cm dishes, starved in serum-free medium for 24 h and then stimulated with 10 % fetal bovine serum for 2 h. Then, the cells were lysed in a buffer containing 20 mM Tris–HCl, pH 7.6, 100 mM NaCl, 1 % Triton-X-100, 10 mM MgCl_2_, 2 mM NaF, and protease inhibitors. The lysates were clarified and the protein concentrations were determined by the bicinchoninic acid protein assay (Thermo Fisher Scientific, Waltham, MA, USA) and normalized. Cell lysates of HeLa cell lines were treated with GTP or GDP and used as positive or negative controls to assess the validity of the pull-down assay [38, 39]. The amounts of GTP-bound RhoA, Cdc42 and Rac1 in equal amounts of total protein were enriched and extracted using their affinity for the downstream effector proteins. The pull-downs (active RhoA, Cdc42 or Rac1) and cell extracts (total RhoA, Cdc42 or Rac1) were analyzed by SDS-PAGE followed by Western blotting with a RhoA-, Cdc42- or Rac1-specific antibody, respectively. Proteins were separated by 4–15 % SDS-polyacrylamide gel electrophoresis (SDS-PAGE) and electroblotted on to polyvinylidene difluoride membranes. After blocking, the membranes were probed with primary antibodies against RhoA, Cdc42 and Rac1. Subsequently, after incubation with horseradish peroxidase-conjugated secondary antibodies, the antigen-antibody complexes were visualized using enhanced chemiluminescence (Thermo Fisher Scientific). Images were captured using an image analyzer (LAS 3000; Fuji Film, Tokyo, Japan).

### Western blotting

Pancreatic cancer cells were lysed using a radioimmunoprecipitation assay buffer (Thermo Fisher Scientific) containing protease inhibitors and phosphatase inhibitors. The lysates were clarified and the protein concentrations were determined by the bicinchoninic acid protein assay (Thermo Fisher Scientific) and normalized. Proteins were separated by 4–15 % SDS-polyacrylamide gel electrophoresis (SDS-PAGE) and electroblotted on to polyvinylidene difluoride membranes. After blocking in 3%FBS in wash buffer (0.2 M Tris-HCl, pH 7.6, 1.5 M NaCl, 0.1 % Tween 20), the membranes were incubated with the specific primary antibodies against ARHGEF15 (Santa Cruz Biotechnology, Dallas, TX, USA) diluted in blocking solution, at the appropriate dilutions. To ensure equal loading, the membranes were probed with anti-β-Actin antibody (Sigma-Aldrich). The membranes were then washed five times, incubated at room temperature for 1 h with a secondary antibody diluted in 5 % non-fat milk in wash buffer. After an additional five washes, the proteins were detected using ECL Western Blotting Detection Reagent, in accordance with the manufacturer’s protocol.

### Stealth RNA-mediated interference and overexpression assays

Cells were transfected with stealth RNA-mediated interference (RNAi; Life Technologies) for ARHGEF15, RhoA or stealth RNAi negative control (Life Technologies) using Lipofectamine RNAiMAX (Life Technologies), in accordance with the manufacturer’s protocol. The Halo-tagged ARHGEF15 expression vector pFN21A was transfected using the Viafect transfection kit (Promega), in accordance with the manufacturer’s protocol. All the experiments, both those involving downregulation of ARHGEF15/RhoA, and those with overexpression of ARHGEF15 were carried out using transient assays.

### Quantitative real-time reverse-transcriptase PCR quantification

Total RNA was isolated from the cells using the RNeasy Mini kit (Qiagen, Venlo, Netherlands). For the clinical PDAC analysis, total RNA was extracted from the tumor area using the Arcturus Paradise PLUS Whole Transcript Reverse Transcription Kit (Life Technologies), in accordance with the manufacturer’s protocol. The cDNAs were synthesized using the Vilo Reverse Transcription kit (Life Technologies). Reactions were carried out using the TaqMan Gene Expression Master Mix (Life Technologies) in an ABI Prism 7900 platform (Life Technologies), in accordance with the manufacturer’s protocol. ACTB was used to normalize the gene expressions (ΔCt) and 2^−ΔΔCt^ to calculate the mRNA expression levels. The following primers and probe sets were used to analyze the respective genes: Hs01060665_g1 for ACTB, Hs00209087_m1 for ARHGEF15, Hs00938813_m1 for SEPT6, Hs00251579_m1 for MATR3 and Hs01056041_m1 for PMSE4 (Life Technologies). If the Ct value of ACTB in real-time RT-PCR was less than 35, the data were used to calculate the gene expression level. The relative quantification of ARHGEF15 was performed using the comparative cycle threshold method.

### Statistical analysis

Statistical analysis to determine the significances of differences in the proliferative activity and motility of the cells was conducted using a two-tailed Student’s *t*-test. *P*-values of <0.05 were considered as denoting statistical significance. Error bars represent standard deviations.

## Additional files

Additional file 1: Genes significantly correlated with the OS in the PDAC patients. (PDF 70 kb) Additional file 2: Figure S1. Higher expression of HNF1B is correlated with worse survival. (PDF 39 kb) Additional file 3: Figure S2. ARHGEF15 contributes to cellular proliferation. ** a** Relative cellular proliferation and RhoA activation after knockdown of ARHGEF15 in Hs766T cells. **b **Relative cellular proliferation and RhoA activation in response to ARHGEF15 overexpression in  MIAPaCa-2. (PDF 135 kb)

## Abbreviations

ARHGEF15: Rho guanine nucleotide exchange factor 15
FFPE: formalin-fixed, paraffin-embedded
GEF: guanine nucleotide exchange factor
OS: overall survival
PDAC: pancreatic ductal adenocarcinoma
