# Supplementary material for: ARHGEF15 overexpression worsens the prognosis in patients with pancreatic ductal adenocarcinoma through enhancing the motility and proliferative activity of the cancer cells
Source: Mol Cancer. 2016 May 4;15:32. doi: 10.1186/s12943-016-0516-4 (PMC4857279; doi:10.1186/s12943-016-0516-4)
Supplement: Additional file 1: — Genes significantly correlated with the OS in the PDAC patients. (PDF 70 kb) [file 12943_2016_516_MOESM1_ESM.pdf]

ranking	ps	gene	name	p value	median of high expression	median of low expression	prognosis
1	g6031204_3p_at	HNF1B	HNF1 homeobox B	0.000002	413	715	poor
2	g7662377_3p_at	ARHGEF15	Rho guanine nucleotide exchange factor (GEF) 15	0.000025	420.5	786	poor
3	228012_3p_at	MATR3	matrin 3	0.000033	381	755	poor
4	Hs.90998.3.S1_3p_a_at	SEPT6	septin 6	0.000045	413	640	poor
5	Hs.112396.0.S1_3p_at	PSME4	proteasome (prosome, macropain) activator subunit 4	0.000051	413	755	poor
6	206147_3p_x_at	SCML2	sex comb on midleg-like 2 (Drosophila)	0.000057	428	629	poor
7	Hs2.293216.1.S1_3p_s_at	PPIL4 /// ZC3H12D	peptidylprolyl isomerase (cyclophilin)-like 4 /// zinc finger CCCH-type containing 12D	0.000086	436	755	poor
8	Hs.278657.1.S1_3p_a_at	MST1 /// MST1P2 /	macrophage stimulating 1 (hepatocyte growth factor-like) /// macrophage stimulating 1 (hepatocyte grow	0.000104	367	715	poor
9	g4417209_3p_a_at	ASH2L	ash2 (absent, small, or homeotic)-like (Drosophila)	0.000106	428	640	poor
10	g8923098_3p_s_at	TRPM6	transient receptor potential cation channel, subfamily M, member 6	0.000116	786	442.5	good
11	Hs.105104.0.A1_3p_x_at	RASSF6	Ras association (RalGDS/AF-6) domain family member 6	0.000188	449	826	poor
12	Hs2.383169.1.S1_3p_at	IGHA1 /// IGHD ///	immunoglobulin heavy constant alpha 1 /// immunoglobulin heavy constant delta /// immunoglobulin hea	0.000240	786	442.5	good
13	Hs2.321733.1.S1_3p_x_at	LOC100292680	hypothetical LOC100292680	0.000242	428	715	poor
14	Hs.129061.0.S1_3p_at	C16orf88	chromosome 16 open reading frame 88	0.000258	449	715	poor
15	Hs.101689.0.A1_3p_at	RBMS3	RNA binding motif, single stranded interacting protein 3	0.000295	428	640	poor
16	g11321627_3p_at	CTRC	chymotrypsin C (caldecrin)	0.000307	413	640	poor
17	Hs.5794.0.A1_3p_at	BRP44	brain protein 44	0.000329	786	431	good
18	Hs.124776.0.A1_3p_at	CDH6	cadherin 6, type 2, K-cadherin (fetal kidney)	0.000375	413	640	poor
19	g5729872_3p_a_at	HPSE	heparanase	0.000377	786	449.5	good
20	Hs.101651.0.S1_3p_at	KRT8P12	keratin 8 pseudogene 12	0.000380	436	715	poor
21	Hs.78353.2.A1_3p_a_at	SRPK2	SRSF protein kinase 2	0.000391	644	432	good
22	Hs.5905.0.A1_3p_at	GABPA	GA binding protein transcription factor, alpha subunit 60kDa	0.000412	449	755	poor
23	Hs2.385557.1.S1_3p_at	SORBS2	sorbin and SH3 domain containing 2	0.000452	449	640	poor
24	Hs.98288.0.A1_3p_at	AQPEP	laeverin	0.000455	614	397	good
25	Hs.303157.10.S1_3p_x_at	PRSS3	protease, serine, 3	0.000463	449	640	poor
26	Hs.7655.2.A1_3p_a_at	U2AF2	U2 small nuclear RNA auxiliary factor 2	0.000474	367	625	poor
27	Hs.23439.0.A1_3p_at	C4orf32	chromosome 4 open reading frame 32	0.000496	449	640	poor
28	Hs2.272321.2.S1_3p_at	TTC26	tetratricopeptide repeat domain 26	0.000508	866	442.5	good
29	g7019406_3p_a_at	MGAT4C	mannosyl (alpha-1,3-)-glycoprotein beta-1,4-N-acetylglucosaminyltransferase, isozyme C (putative)	0.000567	413	625	poor
30	Hs.202737.1.A1_3p_at	WDR89	WD repeat domain 89	0.000591	786	443	good
31	Hs.173048.0.S1_3p_at	LOC100268168	hypothetical LOC100268168	0.000606	614	420.5	good
32	g4506144_3p_x_at	PRSS1	protease, serine, 1 (trypsin 1)	0.000608	449	640	poor
33	Hs2.16758.1.A1_3p_s_at	SPIRE1	spire homolog 1 (Drosophila)	0.000621	413	715	poor
34	g13543517_3p_s_at	CELA3A /// CELA3[illegible]	chymotrypsin-like elastase family, member 3A /// chymotrypsin-like elastase family, member 3B	0.000670	449	640	poor
35	Hs.16340.0.S2_3p_at	SUOX	sulfite oxidase	0.000709	413	640	poor
36	g4505846_3p_s_at	PLA2G1B	phospholipase A2, group IB (pancreas)	0.000740	449	640	poor
37	Hs.40323.2.A1_3p_at	BUB3	Budding uninhibited by benzimidazoles 3 homolog (yeast)	0.000809	436	700	poor
38	g12803670_3p_a_at	DUSP4	dual specificity phosphatase 4	0.000814	644	442.5	good
39	g3387924_3p_a_at	RNF13	ring finger protein 13	0.000821	436	625	poor
40	g180196_3p_s_at	CD44	CD44 molecule (Indian blood group)	0.000833	636	442.5	good
41	Hs.69171.3.A1_3p_at	PKN2	protein kinase N2	0.000835	644	432	good
42	g7706400_3p_a_at	TAOK2	TAO kinase 2	0.000838	413	625	poor
43	g6118538_3p_a_at	BACE1	beta-site APP-cleaving enzyme 1	0.000873	413	628	poor
44	Hs.156346.0.S1_3p_a_at	TOP2A	topoisomerase (DNA) II alpha 170kDa	0.000880	786	424.5	good
45	Hs.129872.1.S2_3p_at	SPAG9	sperm associated antigen 9	0.000946	786	449.5	good
46	g4505802_3p_x_at	PIK3CG	phosphoinositide-3-kinase, catalytic, gamma polypeptide	0.000959	449	625	poor
