# Supplementary figures and images for: ARHGEF15 overexpression worsens the prognosis in patients with pancreatic ductal adenocarcinoma through enhancing the motility and proliferative activity of the cancer cells
Source: Mol Cancer. 2016 May 4;15:32. doi: 10.1186/s12943-016-0516-4 (PMC4857279; doi:10.1186/s12943-016-0516-4)

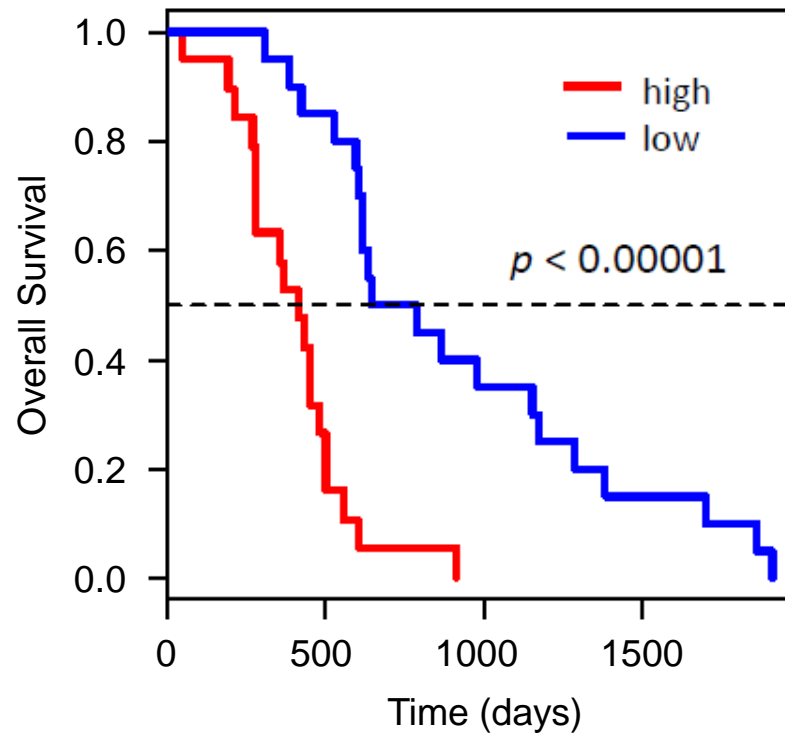

**Additional file 2: Figure S1.** Higher expression of HNF1B is correlated with worse survival.

Supplement: Additional file 2: Figure S1. — Higher expression of HNF1B is correlated with worse survival. (PDF 39 kb) [file 12943_2016_516_MOESM2_ESM.pdf]

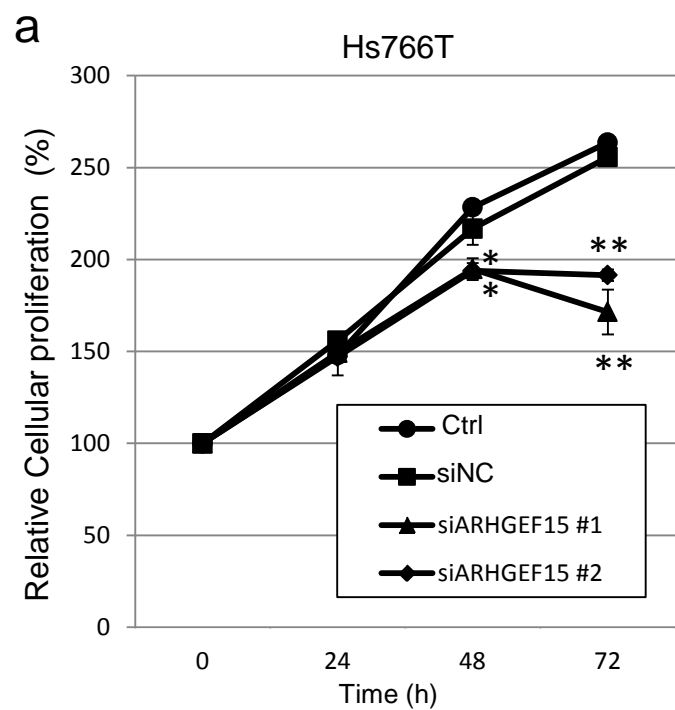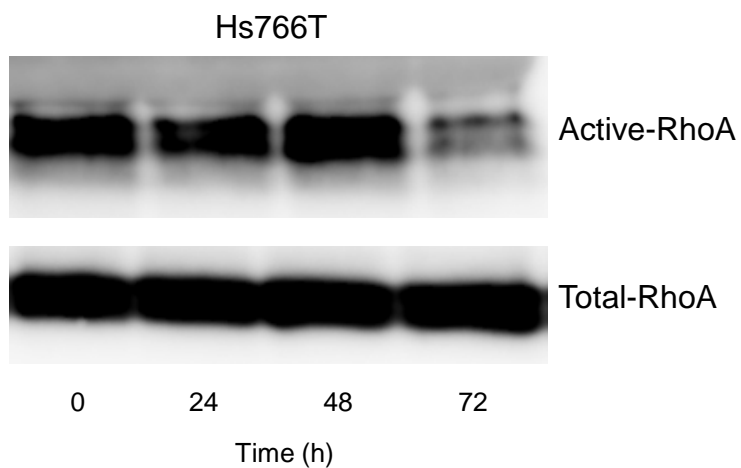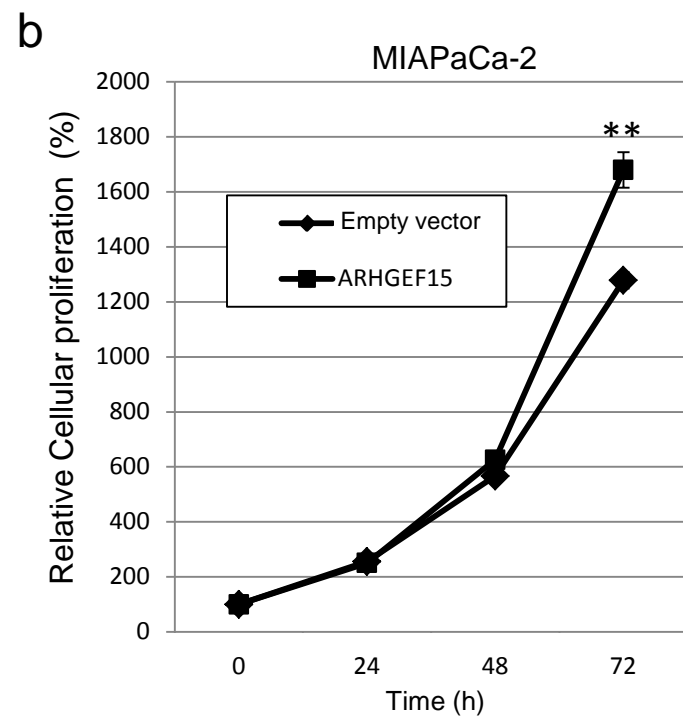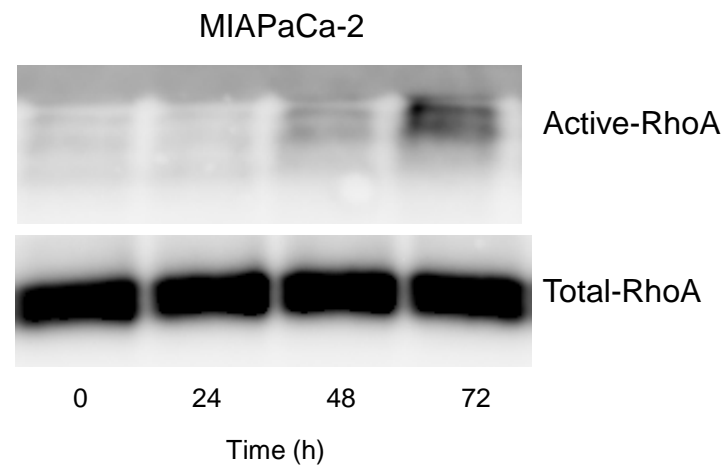

**Additional file 3: Figure S2.** ARHGEF15 contributes to cellular proliferation.

Supplement: Additional file 3: Figure S2. — ARHGEF15 contributes to cellular proliferation. a Relative cellular proliferation and RhoA activation after knockdown of ARHGEF15 in Hs766T cells. b Relative cellular proliferation and RhoA activation in response to ARHGEF15 overexpression in MIAPaCa-2. (PDF 135 kb) [file 12943_2016_516_MOESM3_ESM.pdf]
